# Cardiopulmonary exercise testing in long covid shows the presence of dysautonomia or chronotropic incompetence independent of subjective exercise intolerance and fatigue

**DOI:** 10.1186/s12872-024-04081-w

**Published:** 2024-08-08

**Authors:** Timo Mustonen, Mari Kanerva, Ritva Luukkonen, Hanna Lantto, Arja Uusitalo, Päivi Piirilä

**Affiliations:** 1 Department of Clinical Physiology, Peijas Hospital, HUS Medical Diagnostic Center, Helsinki University Hospital and Helsinki University, Stenbäckinkatu 11 C, PL 281, Helsinki, 00029 Finland; 2 Department of Internal Medicine and Rehabilitation, Helsinki University and Helsinki University Hospital, Helsinki, Finland; 3 Department of Infection Control, Turku University Hospital, The wellbeing services county of Southwest Finland, Turku, Finland; 4 Finnish Institute of Occupational Health, Helsinki, Finland; 5 Department of Clinical Physiology, Park Hospital, HUS Medical Diagnostic Center, Helsinki University Hospital and Helsinki University, Helsinki, Finland; 6 Division of Clinical Physiology and Nuclear Medicine, HUS Medical Diagnostic Center, Helsinki University Hospital and Helsinki University, Helsinki, Finland

**Keywords:** Cardiopulmonary exercise testing, Long covid, Exercise intolerance, Fatigue, Dysautonomia, Sympathetic overactivity, Chronotropic incompetence

## Abstract

**Background:**

After COVID-19 infection, 10–20% of patients suffer from varying symptoms lasting more than 12 weeks (Long COVID, LC). Exercise intolerance and fatigue are common in LC. The aim was to measure the maximal exercise capacity of the LC patients with these symptoms and to analyze whether this capacity was related to heart rate (HR) responses at rest and during exercise and recovery, to find out possible sympathetic overactivity, dysautonomia or chronotropic incompetence.

**Methods:**

Cardiopulmonary exercise test was conducted on 101 LC patients, who were admitted to exercise testing. The majority of them (86%) had been treated at home during their acute COVID-19 infection. Peak oxygen uptake (VO2peak), maximal power during the last 4 min of exercise (Wlast4), HRs, and other exercise test variables were compared between those with or without subjective exercise intolerance, fatigue, or both.

**Results:**

The measurements were performed in mean 12.7 months (SD 5.75) after COVID-19 infection in patients with exercise intolerance (group EI, 19 patients), fatigue (group F, 31 patients), their combination (group EI + F, 37 patients), or neither (group N, 14 patients). Exercise capacity was, in the mean, normal in all symptom groups and did not significantly differ among them. HRs were higher in group EI + F than in group N at maximum exercise (169/min vs. 158/min, *p* = 0.034) and 10 min after exercise (104/min vs. 87/min, *p* = 0.028). Independent of symptoms, 12 patients filled the criteria of dysautonomia associated with slightly decreased Wlast4 (73% vs. 91% of sex, age, height, and weight-based reference values *p* = 0.017) and 13 filled the criteria of chronotropic incompetence with the lowest Wlast4 (63% vs. 93%, *p* < 0.001), VO2peak (70% vs. 94%, *p* < 0.001), the lowest increase of systolic blood pressure (50 mmHg vs. 67 mmHg, *p* = 0.001), and the greatest prevalence of slight ECG-findings (*p* = 0.017) compared to patients without these features. The highest prevalence of chronotropic incompetence was seen in the group N (*p* = 0.022).

**Conclusions:**

This study on LC patients with different symptoms showed that cardiopulmonary exercise capacity was in mean normal, with increased sympathetic activity in most patients. However, we identified subgroups with dysautonomia or chronotropic incompetence with a lowered exercise capacity as measured by Wlast4 or VO2peak. Subjective exercise intolerance and fatigue poorly foresaw the level of exercise capacity. The results could be used to plan the rehabilitation from LC and for selection of the patients suitable for it.

**Supplementary Information:**

The online version contains supplementary material available at 10.1186/s12872-024-04081-w.

## Introduction

In about one fifth of those having had coronavirus disease 2019 (SARS-CoV-2 NhO) infection, variable symptoms continue after the acute phase of the disease [1]. The long-lasting symptoms have been called Long COVID (LC) if no other diagnoses explain the symptoms that usually had occurred 3 months after the initial infection and had lasted for at least 2 months [1, 2]. LC may manifest after severe [2, 3] or mild COVID-19 infections [4]. The most common symptoms are fatigue and exercise intolerance, cognitive disturbances, cough, dyspnea, chest pain, tachycardia, muscle pain, brain fog, depression, or gastric symptoms [1–4], and rehabilitation from these symptoms is a challenge to medical professionals.

Reduced exercise capacity after COVID-19 infection has been studied in athletes, and it has not been found to be systematically associated with structural heart muscle or coronary changes [5, 6]. Good physical fitness could be a protective factor regarding LC, but it does not exclude it [7–9]. Neither are there any pulmonary explanations for LC [10–12]. The lung function problems associated with LC have been regarded as functional or normal recovery from a severe infection [13–15]. Increased tendency to hyperventilation has also been reported in LC patients [16].

Concerning the physiologic phenomena underlying fatigue, dysfunction of the autonomic nervous system [17], sympathetic overactivity, or an imbalance between the sympathetic and parasympathetic functions [17–21] have been discussed. These might explain increased heart rates at rest and during exercise [22] and attenuated recovery of heart rate [23]. According to a meta-analysis, deconditioning, dysfunctional breathing, chronotropic incompetence, and abnormal oxygen extraction have been associated with the LC condition [24].

In our hospital, there is an outpatient clinic for patients suffering from prolonged symptoms after LC. The following criteria are applied: SARS-CoV2 NhO positivity during the acute infection and continuation of the symptoms or the development of new symptoms three months after the initial COVID-19 infection lasting at least two months with no other explanation. [1]. The clinicians working at the LC clinic referred patients to cardiopulmonary exercise testing (CPX) on clinical basis, to assess the true exercise capacity behind the symptoms for working capability reasons and/or to exclude ischemic heart disease or undiagnosed respiratory disease. The present study is a retrospective register study of LC patients studied with CPX.

Aims of the study (1) To assess the objective level of exercise capacity of those with subjective exercise intolerance or fatigue or both (2) To assess the heart rate behavior before, during and after the exercise test as an indication of cardiac autonomic nervous function related to exercise. The hypotheses were that exercise capacity would be low in LC patients who subjectively felt exercise intolerance or fatigue and if so, could autonomic dysfunction or chronotropic incompetence explain the finding.

## Patients

The patient material consisted of patients with LC-symptoms referred from the HUS outpatient clinic because of COVID-19 long-term symptoms for CPX to the unit of clinical physiology at the Park Hospital of Helsinki University Hospital between 1.6.2021–31.12.2022. A total of 106 consecutive LC patients were tested with CPXs during that time. However, data from two patients were discarded due to failure in breath gas analysis, two did not reach a satisfactory level of exertion (respiratory exchange rate, RER, below 1), and one because of an earlier lung resection operation. Thus, a total of 101 patients were included in the study (Table 1**)**. The initial COVID-19 infection had been mild for the majority; only 14% had needed hospitalization. The CPX examinations were performed within the mean of 12.7 (SD5.75; 5–29) months after the onset of a PCR-positive COVID-19 infection.

**Table 1:** The anthropometric, smoking, and spirometry data of the patients in the different symptom groups

	Neither (Group *N*) *n* = 14	Fatigue (Group F) *n* = 31	Exercise Intolerance (Group EI) *n* = 19	Combination (Group EI + F) *n* = 37	*p*-value
Sex M/F (%)	5/9 (56%)	12/19 (63%)	7/12 (58%)	14/23 (61%)	
Age (years) Mean (SD)	45.7 (11.1)	42.9 (9.3)	47.2 (6.8)	43.4 (10.6)	0.392
Height (cm); Mean (SD)	172.2 (7.7)	170.2 (6.9)	169.0 (8.1)	174.1 (11.4)	0.176
Weight (kg) Mean (SD)	76.0 (8.9)	74.3 (14.7)	88.0 (23.4)	87.1 (19.8)	0.009
BMI (kg/m^2^)	25.8 (3.7)	25.6 (4.6)	30.5 (6.1)	28.8 (6.4)	0.008
Smokers/ex-smokers (numbers) No/ex/smoker	5/5/4	24/6/1	14/5/0	25/6/6	
FVC (L) Mean (SD)	4.0 (0.9)	4.2 (1.0)	3.9 (0.8)	4.4 (1.4)	0.435
FVC (% pred.)# Mean (SD)	89.9 (11.9)	94.1 (11.7)	92.8 (11.3)	93.1 (12.3)	0.749
FEV1 (L) Mean (SD)	3.2 (0.7)	3.3 (0.7)	3.3 (0.7)	3.6 (1.1)	0.564
FEV1 (% pred.)# Mean (SD)	100.1 (31.7)	94 (10.9)	97.6 (12.7)	95.4 (11.6)	0.650
FEV1/FVC (%)Mean (SD)	81.6 (6.7)	80.2 (5.8)	83.0 (3.2)	81.4 (5.2)	0.369
FEV1/FVC (% pred.)# Mean (SD)	103.4 (8.5)	100.8 (7.3)	105.3 (4.8)	102.7 (6.8)	0.167

Kainu et al.2016 [25] #

In the analysis, we included data from the exercise tests, and physicians’ referrals to the tests, i.e., information regarding actual diseases, medication, initial COVID-19 infection, hospital treatment, and reason for the referral (Supplementary Table 1). Ongoing infective processes and other obvious explanations for the symptoms had been excluded by the referring clinicians.

For comparisons, patients were divided according to the reason of referral to CPX into 4 groups: (1) exercise intolerance (exercise capacity subjectively lower than before COVID-19 disease; group EI, 19 patients). (2) fatigue (sensation of being exhausted in everyday life; group F, 31 patients), those with both exercise intolerance and fatigue (37 patients; group EI + F), and those with neither of them (14 patients; group N) (Supplementary Table 1).

### Methods

A maximal cardiopulmonary exercise test (CPX) was performed using the CPX equipment system (Vyntus CPX, by SensorMedics, Yorba Linda, CA, USA). After spirometry measurements, the patient rested for approximately 10 min in a supine position. Breath gas recording/sampling was started when the patient was sitting on the bicycle. The (inspiratory and expiratory) breath gas recordings/sampling breath by breath continued throughout the CPX, and 30-second mean values of breath gases were reported, the main measured variables being maximal oxygen uptake (VO2peak), CO_2_ production (VCO2), and minute ventilation (VE) and their derivatives.

The CPXs were performed using an electrically braked cycle ergometer (Ergoselect 200P, Ergoline Gmbh, Bitz, Germany). The starting workload was 40 W for women and 50 W for men, and the load was increased at 3-minute intervals by 40–50 W, respectively. For those reporting short walking distances, 20 W loads at 2-minute intervals were used. The exercise was continued until the subjective hard exertion (17–20/20 scale of perceived exertion) and a respiratory exchange rate (RER = VCO2/VO2) of at least 1.0 were reached, and the heart rate goal (HRmaxpred; 205 − 0.5*age) was > 80%. The principal subjective reason or reasons for exercise termination were registered. The forced expiratory volume in 1 s (FEV1) was measured after the first minute of recovery when the patient was still sitting on the bicycle, and then at 4–5 min and at 10 min after the exercise. The patients were in a supine position between the FEV1 measurements. The first ventilatory threshold (AT) was assessed at the point when VCO2, partial pressure of end tidal O2 (PetO2), and ventilatory equivalent for oxygen uptake (VE/VO2) increased related to VO2. A 12-lead ECG was continuously monitored during the exercise and recorded using a computerized software (CardioSoft version 7-7.0851, GE Medical Systems, Milwaukee, WI, USA) with manual blood pressure measurements using a stethoscope and a sphygmomanometer (Erka, Germany). Peripheral arterial oxygen saturation (SpO2) was measured with two pulse oximeters (MySignS, EviteC, NJ, USA), one attached to the subject’s earlobe and the other to the left middle finger.

Heart rate values (HR) were studied before, during and after exercise as follows: (1) supine (HRrest), (2) sitting on the bicycle (HRsitting), (3) sitting on the bicycle just at the start of exercise (HRstart), (4) at maximum exertion (HRmax), and (5) during the recovery phase: at 1 min (HR1min), 2 min (HR2min), 3 min (HR3min), 5 min (HR5min), and 10 min (HR10min).

The normal exercise capacity was defined as maximal power during the last 4 min of exercise (Wlast4) ≥ 80% of the predicted value [26] or oxygen uptake ≥ 80% of the predicted value [27]. To detect abnormally increased HR (i.e. dysautonomia), the triad of resting HR > 75 bpm, HR increase with exercise < 89 bpm, and HR recovery < 25 bpm 1 min after exercise was used [23, 28]. To find out an abnormally low HR reaction (i.e. chronotropic incompetence), we used the following criteria: The maximal VO2 predicted < 85% of predicted value and the age-related HR increase lowered (< 80%), calculated as (HRmax - HRrest)/HR reserve (where HR reserve = HRmax pred - HRrest) without alternative explanations for exercise limitation [29–31]. The CPX results of patients with dysautonomia, or chronotropic incompetence were compared to the results of those patients without these features.

### Statistics

For continuous spirometry variables, symptom groups were compared using the Student’s t-test or Kruskal-Wallis test and the Chi-square test for categorical variables.

We built general linear models when examining the associations among cardiopulmonary exercise tests or heart rates (outcome variables) and symptoms (exercise intolerance or fatigue or both). Only the p-values of the F-test for symptom groups have been presented in tables. Our model building strategy was as follows. First, we computed an unadjusted model. Then, we estimated an adjusted model usually using age, BMI, and sex as variables, but the adjustments varied and were dependent on the outcome variables, which are given in the headers or footnotes of the tables. The maximal heart rates were adjusted to HRrest, and the heart rates of the recovery phase also to the achieved HRmax. The comparisons among some variables were computed using the Chi-Square test.

A p-value of < 0.05 was considered statistically significant. All analyses were carried out using SPSS (version 27) program.

Users of beta-blockers were excluded from HR calculations expect for those who had had at least a two-day break in their use.

## Results

Among the 101 patients included in the study, 56 felt subjective exercise intolerance (group EI) and 68 felt fatigue (group F). Among these patients, 37 had both symptoms (group EI + F) and a minority, 14 had neither of them (group N). A variety of other symptoms were present as well, but comorbidities were not common (Supplementary Table 1). Three patients had obstruction seen as FEV1/VC < 0.7, one was earlier diagnosed with COPD. Two patients had restriction with lowered diffusing capacity, one of them with associated atelectasis after COVID-19 infection. On demand or regular beta-blockers were used by 23 subjects and asthma medication by 15 subjects. The clinicians considered that neither these comorbidities nor the medications explained the LC-symptoms of the patients. The analyses showed that asthma or the use of asthma medication did not influence the results (see the comment on the use of beta-blockers in the previous chapter.

The most common reasons for terminating the exercise were subjective overall or leg fatigue (76 out of 101 patients) (Supplementary Table 1). All of those included in the study had RER > 1 (Table 2), and in 82 patients, it was ≥ 1.1 (81%). In one case, an ischemic ECG reaction and chest pain were reported, and in another case, chest pain alone was reported. In the FEV1 follow-up, the FEV1 variation was < 12%, except for one subject, 15%, with diagnosed asthma.

**Table 2:** The results of CPX in peak exercise for those with exercise intolerance (EI), fatigue (F), combination (EI + F), and neither (N). Blood pressure values for rest, peak and recovery phases are also provided

	Group *N* *n* = 14 Mean (SD)	Group F *n* = 31 Mean (SD)	Group EI *n* = 19 Mean (SD)	Group EI + F *n* = 37 Mean (SD)	adjusted
Borg Subjective Scale (6–20)	18.4 (1.4)	19.2 (0.9)	18.4 (1.2)	18.1 (1.3)	0.005 1)
RER (Respiratory Exchange Rate)	1.14 (0.07)	1.17 (0.09)	1.13 (0.07)	1.16 (0.06)	0.278 1)
Wlast4 (Maximal power during the last 4 min of exercise) (W)	121.9 (36.2)	133.2 (43.8)	140.1 (42.8)	138.5 (50.1)	0.571 1)
Wlast4 (% pred.) *	83.1 (25.4)	88.9 (25.6)	90.9 (17.1)	84.7 (25.2)	0.708 2)
Wlast4 (% pred) < 80% of predicted	8 (57%)	10 (32%)	5 (26%)	17 (46%)	0.210 #
VO2peak (Maximal oxygen consumption) (ml/min)	1844.6 (541.0)	1937.2 (518.2)	2135.0 (615.6)	2092.0 (595.2)	0.396 1)
VO2peak (% pred.) **	84.5 (18.4)	85.8 (19.3)	96.7 (17.4)	92.2 (18.3)	0.124 2)
VO2kgpeak (VO2peak/min/kg) (ml/min/kg)	24.4 (6.0)	26.5 (6.4)	24.4 (4.1)	24.7 (7.2)	0.679 4)
VO2kgpeak (% pred) ** (% pred.)	80.2 (19.2)	83.2 (19,2)	81.3 (14.8)	78.6 (21.6)	0.810 2)
VO2kgpeak (% pred) < 80% of predicted	8 (57%)	14 (45%)	8 (42%)	17 (46%)	0.847 #
AT (First ventilatory threshold) (% pred.) ***	89.5 (22.8)	96.5 (22.3)	102.6 (18.6)	97.9 (26.1)	0.461 2)
HRmax (% pred.)	85.4 (10.5)	91.3 (8.0)	87.7 (7.2)	91.0 (8.0)	0.031 5)
VO_2_/HR (Oxygen pulse) (% pred.) ***	108.1 (21.2)	105.3 (24.6)	119.8 (25.2)	108.9 (26.9)	0.333 2)
Wmax/VO2peak (Work efficiency) (%)	19.8 (1.7)	20.5 (2.8)	19.6 (3.0)	19.7 (1.9)	0.742 1)
Breathing Reserve (% MVV)	41.1 (16.7)	39.8 (14.6)	32.3 (18.5)	34.1 (16.7)	0.657 1)
VE/VO_2_ (Ventilatory equivalent for O2) (% pred.) **	124.5 (22.6)	126.2 (20.2)	125.9 (20.4)	135.0 (27.2)	0.315 3)
VE/CO_2_ (Ventilatory equivalent for CO2) (% pred.) **	121.3 (18.2)	118.6 (147.8)	122.9 (17.9)	129.1 (24.4)	0.210 3)
FetCO_2_ (Fraction of endtidal CO2) (%)	4.9 (0.5)	4.8 (0.5)	4.7 (0.6)	4.6 (0.7)	0.327 1)
Breathing Frequency (1/min)	34.2 (7.9)	33.8 (8.6)	37.5 (8.3)	36.8 (8.6)	0.654 1)
VE/VCO2 slope	27.5 (2.3)	27.5 (2.7)	27.6 (3.6)	29.2 (4.5)	0.138 1)
Systolic Blood Pressure at Rest (mmHg)	121.64 (22.4)	121.9 (12.2)	123.8 (14.6)	126.84 (15.86)	0.416 1)
Systolic Blood Pressure at Peak (mmHg)	182.71 (30.3)	185.8 (29.2)	191.4 (24.1)	190.9 (24.0)	0.684 1)
Increase of Blood Pressure (mmHg)	64.0 (16.7)	66.1 (25.1)	62.3 (18.4)	65.1 (21.8)	0.741 1)
Systolic Blood Pressure During Recovery Phase (about minutes 3–4)	128.3 (18.8)	143.2 (27.0)	145.3 (20.0)	151.4 (27.0)	0.081 1)

*Arstila et al. 1990 [26], **Seliger et al. 1978 [27], ***Wasserman et al. 1987 [31]

(1) adjusted for sex, age, and BMI, (2) Reference values include sex, age, weight, (3) adjusted for BMI (4) adjusted for age and sex, (5) sex and BMI. # Chi-Square

MVV = maximal voluntary ventilation (FEV1-estimated maximal minute ventilation), Breathing Reserve = measured maximal ventilation / MVV (percentage), % pred. = percent of predicted value

The average maximal power during the last 4 min of exercise (Wlast4%) was 86.9% in the entire patient group (*N* = 101) [26], although 40% of patients had a lowered exercise capacity (< 80% of the predicted value). The mean exercise capacity did not differ among the groups, with the mean values ranging from 83% in group N to 91% in group EI (Table 2). When groups with EI, F or EI + F were compared to group N, the findings did not differ significantly among the groups. (Table 2). Also, the oxygen consumption (VO2peak) was in mean normal (mean values ranging from 85% in group N to 97% in group EI) [27]. In relation to weight, oxygen consumption (VO2kgpeak) was also in mean normal but 47% of the participants had VO2kgpeak < 80% of the predicted value. In groups N, F and EI VO2kgpeak was in mean normal (80%, 83%, 81%, respectively) but was slightly lowered in group EI + F (79%) without significant differences among the groups (Table 2). Systolic blood pressure at rest and peak systolic blood pressure during exercise did not in mean differ among the groups.

**Fig. 1:**
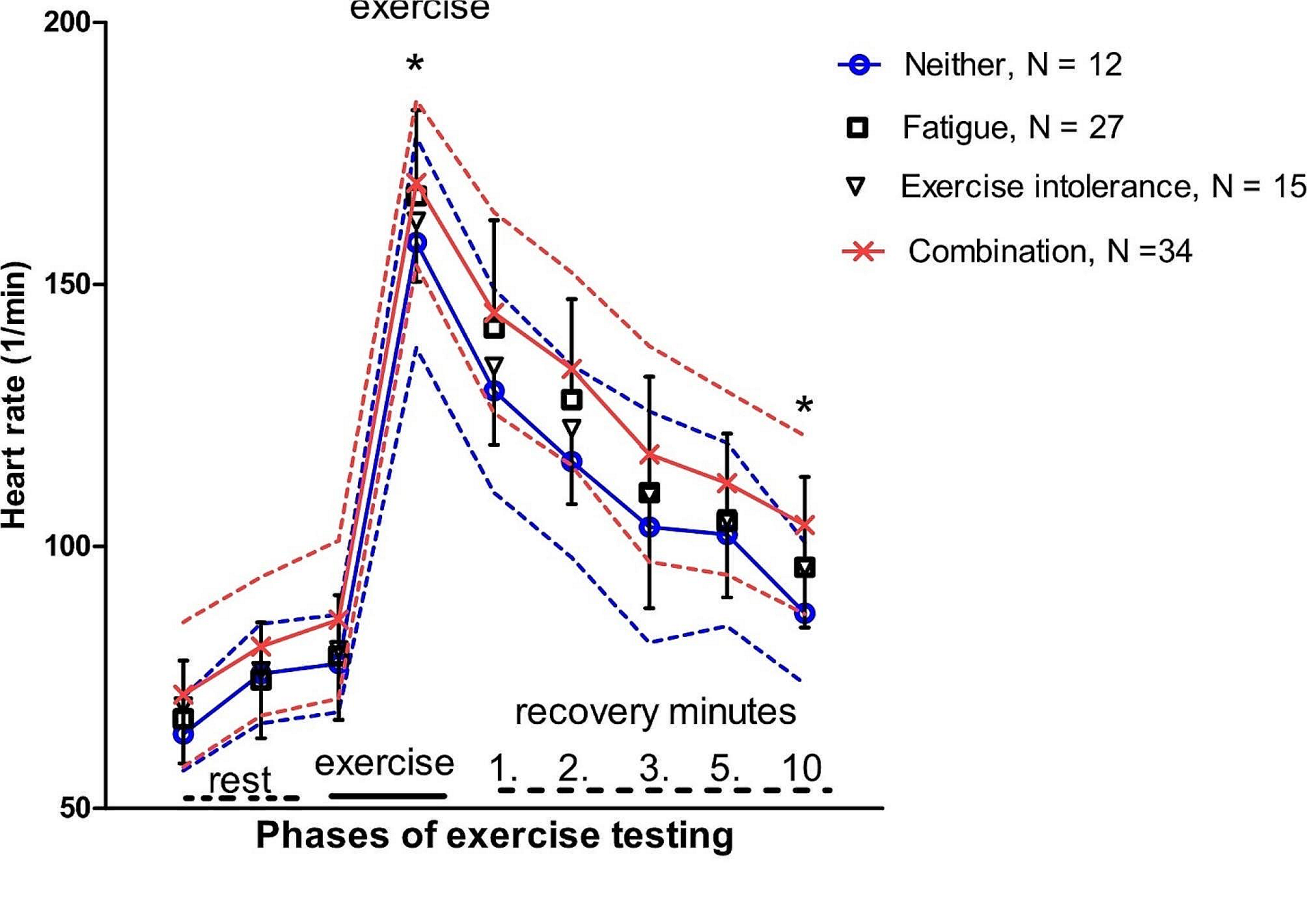
The heart rates associated with CPX testing: pretest time, exercise, and recovery in groups EI, F, EI + F, and group N. Adjustments were performed for BMI, sex, age, and use of beta-blockers at rest. The maximal heart rate was adjusted for BMI, sex, age, use of beta-blockers, and HRrest. In the recovery phase, adjustments were calculated for BMI, sex, age, use of beta-blockers, and maximal heart rate. * Indicates significant difference at *p* < 0.05 level

After excluding the users of beta-blockers, there were 88 patients. To estimate sympathetic overactivity, the heart rates at rest, during exercise, and recovery were analyzed. The relative HR increase (HR increase related to HRreference) (87% vs. 80%, *p* = 0.042) and HRmax of predicted (92% vs. 87%, *p* = 0.022) were higher in group EI + F than in group N (See the adjustments used in Table 3). During recovery, HR10min was higher in group EI + F than in group N (104/min vs. 87/min, *p* = 0.026) (Fig. 1; Table 3).

**Table 3:** The HR comparisons between the LC symptom groups associated with exercise and recovery phase

	Patients				Unadjusted model	Adjusted model			
**Outcome**	**Neither** **(Group N)** *n* = 12	**Fatigue** **(Group F)** *n* = 27	**Exercise Intolerance** **(Group EI)** *n* = 15	**Combination** **(Group EI + F)** *n* = 34	**All** **Patients** *n* = 88	**All** **Patients** *n* = 88	**Group F vs.** **Group N**	**Group EI vs.** **Group N**	**Group EI + F** vs. **Group N**
	Mean (SD)	Mean (SD)	Mean (SD)	Mean (SD)	F-test	F-test	t-test	t-test	t-test
HR supine	64.0 (7.5)	67.1 (11.1)	69.5 (10.9)	71.7 (13.8)	0.214	0.267 *)	0.584 *)	0.159 *)	0.106
HR sitting	75.8 (9.5)	74.5 (11.0)	76.3 (12.9)	80.9 (13.2)	0.192	0.186 *)	0.631 *)	0.578 *)	0.249
HR start	77.6 (9.7)	79.1 (11.6)	80.3 (13.4)	86.0 (15.1)	0.119	0.168 *)	0.917 *)	0.383 *)	0.100
HRincrease (1/min)	94.0 (19.9)	99.7 (16.9)	92.5 (12.7)	97.7 (18.7)	0.562	0.207 1)	0.133 1)	0.148 1)	**0.034**
Relative HRincr.(%)	79.8 (16.2)	85.9 (12.6)	83.1 (10.8)	87.1 (11.7)	0.321	0.241 2)	0.147 2)	0.169 2)	**0.042**
HRmax (% ofpred.)	87.1 (10.4)	91.1 (8.1)	89.6 (6.5)	92.1 (7.22)	0.260	0.150 3)	0.105 3)	0.157 3)	**0.022**
HRmax	158 (20.1)	166.9 (16.4)	162.1 (11.6)	169.4 (15.7)	0.146	0.207 1)	0.133 1)	0.148 1)	**0.034**
**RECOVERY**									
HR1min	129.7 (18.8)	141.7 (20.6)	132.4 (15.0)	144.6 (19.2)	**0.049**	0.359 4)	0.293 4)	0.974 4)	0.187
HR2 min	116.2 (18.3)	128.0 (19.2)	122.5 (14.4)	133.9 (18.4)	**0.022**	0.287 4)	0.209 4)	0.447 4)	0.067
HR 3 min	103.7 (19.4)	110.3 (22.1)	109.7 (21.5)	117.6 (20.6)	0.206	0.882 4)	0.944 4)	0.725 4)	0.623
HR5 min	102.3 (17.5)	104.8 (16.7)	105.3 (15.0)	112.1 (17.5)	0.209	0.575 4)	0.536 4)	0.809 4)	0.693
HR10min	87.3 (13.5)	96.0 (17.3)	96.0 (11.5)	104.1 (17.0)	**0.015**	0.089 4)	0.432 4)	0.140 4)	**0.026**

Beta-blocker users are excluded from the analyses

*) adjusted for sex, age, and BMI

1) adjusted for sex, age, BMI, and HR supine

2) adjusted for sex and BMI

3) adjusted for sex, BMI, and HR supine

4) adjusted for sex, age, and BMI, and HRmax

Beta-blocker users excluded, 12/88 subjects had dysautonomia (Table 4). They had a significantly lower maximal work power (mean 73.2% vs. 90.6% of the predicted value, *p* = 0.017) and work efficiency (Wmax/VO2peak: mean 19.3% vs. 20.3%, *p* = 0.031) compared to those without dysautonomia, but there was no significant difference in oxygen uptake. The patients with dysautonomia were evenly spread among the symptom groups (Table 4). There were also 13/88 patients with chronotropic incompetence (Table 5) who had lowered Wlast4 (mean 62.6% vs. 92.7%, *p* < 0.001), a lowered VO2peak (mean 69.9% vs. 93.5%, *p* < 0.001) [27] and a lowered Wmax/VO2peak (mean 18.4% vs. 20.5%, *p* < 0.001) [31] compared to those patients without chronotropic incompetence. Also, the peak systolic blood pressure, the change between rest and peak systolic blood pressure values, and the recovery values were lower in those with chronotropic incompetence than in those without it (*p* = 0.006, 0.001 and 0.024, respectively). They also had a higher breathing reserve compared to the others (mean 48.1% vs. 34.4%; *p* < 0.001).

**Table 4:** The results of CPX analyzed according to abnormally increased HR response suggesting dysautonomia (*n* = 88). Blood pressure values for these groups are also shown

Outcome	No Dysautonomia (*n* = 76, 86%)	Dysautonomia (*n* = 12, 14%)	*p*-values
**Symptoms**			
Patients with Exercise Intolerance (number)	13	2	*p* = 0.965
Patients with Fatigue (number)	24	3	
Patients with Both Exercise Intolerance and Fatigue (number)	29	5	
Patients with Neither Symptom (number)	10	2	
**Outcomes**	**Mean (SD)**	**Mean (SD)**	**adjusted**
Borg Subjective Scale (6–20)	18.5 (1.26)	18.92 (0.9)	0.165 1)
RER (Respiratory Exchange Rate)	1.16 (0.07)	1.15 (0.8)	0.515 1)
Wlast4 (Maximal power during the last 4 min of exercise) (W)	136,8 (45.5)	126.3 (39.1)	0.068 1)
Wlast4 (% pred.) *	**90.6 (24.0)**	**73.2 (13.7)**	**0.017 2)**
Wlast4 (% pred) < 80% of predicted	**24 (32%)**	**9 (75%)**	**0.004** #
VO2peak (Maximal oxygen consumption) (ml/min)	2011.0 (550.8)	1975.3 (559.6)	0.233 1)
VO2peak (% pred.) **	91.2 (18.4)	82.1 (13.9)	0.110 2)
VO2kgpeak (VO2peak/min/kg) (ml/min/kg)	25.6 (6.3)	23.6 (4.9)	0.187 4)
VO2kgpeak (% pred) **	83.2 (19.2)	73.4 (16.2)	0.099 2)
AT (First ventilatory threshold) (% pred.) ***	98.0 (21.8)	87.7 (20.9)	0.132 2)
HRmax (% pred.)	91.4 (7.6)	86.5 (8.3)	0.163 5)
VO_2_/HR (Oxygen pulse) (% pred.) ***	110. 4 (24.1)	98.3 (17.9)	0.091 2)
Wmax/VO2peak (Work efficiency) (%)	20.3 (2.0)	19.3 (1.4)	**0.031 1)**
Breathing Reserve (% MVV)	36.0 (15.9)	39.2 (15.3)	0.458 1)
VE/VO2 (Ventilatory equivalent for O2) (% pred.) **	128.4 (24.7)	135.5 (20.9)	0.320 3)
VE/CO2 (Ventilatory equivalent for CO2) (% pred.) **	122.7 (20.9)	130.5 (17.7)	0.206 3)
FetCO_2_ (Fraction of end tidal CO2) (%)	4.8 (0.6)	4.5 (0.5)	0.147 1)
Breathing Frequency (1/min)	35.7 (8.6)	35.6 (8.0)	0.947 1)
VE/VCO2 slope	28.6 (3.9)	29.1 (3.0)	0.510 1)
Prevalence of ECG findings	12 (16%)	1 (8%)	0.722 #
Systolic Blood Pressure at Rest (mmHg)	123.4 (14.3)	127.2 (17.5)	0.610 1)
Systolic Blood Pressure at Peak (mmHg)	188.6 (26.2)	189.1 (24.8)	0.494 1)
Increase of Blood Pressure (mmHg)	65.2 (22.0)	61.9 (18.4)	0.235 1)
Systolic Blood Pressure during recovery phase (about minutes 3–4) (mmHg)	144.7 (25.6)	144.2 (30.1)	0.925 1)

Beta-blocker users are excluded from the analyses

MVV = maximal voluntary ventilation (FEV1-estimated maximal minute ventilation), Breathing Reserve = measured maximal ventilation / MVV (percentage), % pred. = percent of predicted value. *Arstila et al. 1990 [26], **Seliger et al. 1978 [27], ***Wasserman et al. 1987 [31]

(1) adjusted for sex, age, and BMI, (2) Reference values include sex, age, weight, (3) adjusted for BMI (4) adjusted for age and sex, (5) sex and BMI. # Chi-Square testing

**Table 5:** The CPX results analyzed according to HR criteria for chronotropic incompetence (*n* = 88). Blood pressure values for these groups are also shown

Outcome	No Chronotropic Incompetence (*n* = 75, 85%)	Chronotropic Incompetence (*n* = 13, 15%)	*p*-values
**Symptoms**			
Patients with Exercise Intolerance (number)	12	3	*p* = 0.022 #
Patients with Fatigue (number)	24	3	
Patients with Both Exercise Intolerance and Fatigue (number)	32	2	
Patients with Neither Symptom (number)	7	5	
**Outcomes**	**Mean (SD)**	**Mean (SD)**	**adjusted**
Borg Subjective Scale (6–20)	18.53 (1.25)	18.85 (1.07)	0.366 1)
RER (Respiratory exchange rate, VCO2/VO2)	1.16 (0.07)	1.14 (0.07)	0.101 1)
Wlast4 (Maximal power during the last 4 min of exercise) (W)	142.1 (42.5)	96.8 (37.6)	**< 0.001 1)**
Wlast4 (% pred.) *	**92.7 (21.6)**	**62.6 (17.7)**	**< 0.001 2)**
Wlast4 (% pred) < 80% of predicted	**20 (27%)**	**13 (100%)**	**< 0.001 #**
VO2peak (Maximal oxygen consumption) (ml/min)	**2082.3 (535.5)**	**1566.9 (413.1)**	**< 0.001 1)**
VO2peak (% pred.) **	**93.5 (16.8)**	**69.9 (10.1)**	**< 0.001 2)**
VO2kgpeak (VO2peak/min/kg) (ml/min/kg)	**26.2 (5.9)**	**20.3 (5.5)**	**< 0.001 4)**
VO2kgpeak (% pred)	**84.6 (18.5)**	**65.9 (14.6)**	**0.001 2)**
AT (First ventilatory threshold) (% pred.) ***	98.1 (21.5)	88.2 (23.0)	0.128 2)
HRmax (% pred.)	**92.5 (6.6)**	**80.2 (6.8)**	**< 0.001 5)**
VO_2_/HR (Oxygen pulse) (% pred.) ***	**110.8 (23.3)**	**96.5 (16.5)**	**0.037 2)**
Wmax/VO2peak (Work efficiency) (%)	**20.5 (1.58)**	**18.4 (3.0)**	**< 0.001 1)**
Breathing Reserve (% MVV)	**34.4 (16.1)**	**48.1 (6.9)**	**< 0.001 1)**
VE/VO_2_ (Ventilatory equivalent for O2) (% pred.) **	129.9 (24.6)	126 (17.7)	0.502 3)
VE/CO_2_ (Ventilatory equivalent for CO2) (% pred.) **	123.4 (21.4)	122.8 (15.3)	0.901 3)
FetCO_2_ (Fraction of end tidal CO2) (%)	4.7 (0.6)	4.8 (0.4)	0.722 1)
Breathing Frequency (1/min)	36.5 (8.6)	31.4 (6.0)	0.069 1)
VE/VCO2 slope	28.3 (3.8)	28.0 (3.7)	0.830 1)
Prevalence of ECG-findings	**6 (8%)**	**4 (31%)**	**0.017 #**
Systolic Blood Pressure at Rest (mmHg)	123.9 (15.3)	123.9 (11.1)	0.934 1)
Systolic Blood Pressure at Peak (mmHg)	**191.3 (25.3)**	**173.5 (24.9)**	**0.006 1)**
Increase of Blood Pressure (mmHg)	**67.4 (21.1)**	**49.62 (17.2)**	**0.001 1)**
Systolic Blood Pressure During Recovery Phase (about minutes 3–4)	**147.2 (26.4)**	**130.1 (18.2)**	**0.024 1)**

Beta-blocker users are excluded from the analyses

MVV = maximal voluntary ventilation (FEV1-estimated maximal minute ventilation), Breathing Reserve = measured maximal ventilation / MVV (percentage), % pred. = percent of predicted value. *Arstila et al. 1990 [26], **Seliger et al. 1978 [27], ***Wasserman et al. 1987 [31]

(1) adjusted for sex, age, and BMI, (2) Reference values include sex, age, weight, (3) adjusted for BMI (4) adjusted for age and sex, (5) sex and BMI, # Chi-Square testing

10 out of 88 (11%) patients showed slight ST-depressions associated with exercise testing, usually ranging from 1 to 2 mm; some of these depressions were suspected to be related to sympathetic overactivity, and some aroused a suspicion of ischemia of cardiac muscle (Supplementary Table 1). One of the patients with ST-depressions was found to have dysautonomia and four filled the criteria of chronotropic incompetence (Tables 4 and 5). Thus, in those patients with chronotropic incompetence, the prevalence of the changes in ECG was higher (4/13, 31%) than in the subjects without chronotropic incompetence when beta-blocker users were excluded (6/75, 8%) (*p* = 0.017 Chi-Square test; Table 5). However, the prevalence of ECG-changes did not differ among the symptom groups (Supplementary Table 1).

Among the 13 patients with chronotropic incompetence, five suffered from LC-symptoms other than fatigue or exercise intolerance. Of these, three had severe symptoms and were hospitalized during acute COVID-19, one treated in the intensive care and developed polyneuropathy, while the other two were treated in wards. However, the other 10 patients fulfilling the criteria of chronotropic incompetence had been treated at home during the acute phase of the disease.

Although the other reported symptoms including respiratory symptoms (43 patients) or palpitations (37 patients) were common, only 9 patients felt that respiratory symptoms and 6 that palpitations were the terminating symptoms of the exercise (Supplementary Table 1).

We performed active orthostatic testing for 20 patients, and in 9 (45%) of them, heart rate increased by ≥ 30 beats/minute as a response to upright posture (data not shown).

## Discussion

We studied the association of two main LC-symptoms, exercise intolerance, and fatigue with an objectively measured exercise capacity. We found, unexpectedly, that neither the mean exercise capacity nor the mean oxygen uptake percentage of the predicted value differed among those with or without subjective exercise intolerance or fatigue or those with a combination of these symptoms. Beta-blocker users excluded, 14% fulfilled the criteria of dysautonomia with slightly lowered exercise capacity and work efficiency. Another 15% fulfilled the criteria of chronotropic incompetence, and they had in mean a moderately lowered exercise capacity and a slightly lowered oxygen uptake and work efficiency in comparison to the subjects without these features. This may suggest a poorer aerobic physical performance during exercise and could be considered in expectations of recovery and help in patient selection and designing the rehabilitation.

Before the LC era, chronotropic incompetence has been seen as predictor of cardiovascular disease and mortality [32, 33]. Although the criteria of chronotropic incompetence vary in literature, it has been reported that 1/3 of patients with heart failure would fulfill this criterion and would be associated with poor quality of life and prominent exertional symptoms [29]. There are several reports on chronotropic incompetence being associated with LC, both in hospitalized and non-hospitalized LC patients. Cardiac functional or structural abnormalities associated with the LC condition probably explain the low HR response [24, 29, 30, 34–37], smoking and earlier cardiac diseases have been seen as risk factors for chronotropic incompetence in LC [30]. Earlier studies have shown that the maximal VO2 is lower in those who have suffered from a more severe LC disease [38, 39]. Here, as reported above, only 3 of those with chronotropic incompetence had been hospitalized and the other 10 had been treated at home at the acute phase of the LC disease suggesting that chronotropic incompetence does not only develop in those with severe LC disease.

We recognized 13 patients fulfilling the criteria for chronotropic incompetence [29, 30]. They had the lowest oxygen uptake in the mean, 70% of the predicted value, and the lowest Wlast4 (in the mean, 63% of the predicted value), which is in line with earlier studies on chronotropic incompetence [29, 32, 33]. They also had the lowest blood pressure increase during exercise and during the recovery phase which might suggest a lowered cardiac capacity. The higher breathing reserve, despite the RER values similar to the other subjects, suggests that neither breathing problems nor the submaximal exercise level explain the lowered exercise capacity in the subjects with chronotropic incompetence. Instead, possible cardiac limiting factors might play a role in exercise intolerance in this patient group [36, 40, 41], and in the present study, the greatest percentage of ECG findings suggesting ischemia or sympathetic stimulation were found in those with chronotropic incompetence. However, in the present register study the further development of the disease cannot be followed up.

Although the term dysautonomia refers to varying forms of autonomic dysfunction [17, 41], the HR triad used here as the criterion for dysautonomia has also been used in earlier LC studies, and it has been suggested that dysautonomia explains the fatigue symptoms or functional limitations in LC patients [23, 28, 34, 40]. In the present study, twelve subjects fulfilled the criteria of dysautonomia, with increased resting HR, reduced increase of HR from the high resting level, and slow HR recovery. These patients showed slightly lowered maximal working power and working efficiency (Wmax/VO2peak), which is in line with the findings of an earlier study [23]. The results of some earlier studies suggest that dysautonomia is a mild and reversible condition [17, 23].

In the present study, in group EI + F the maximal HR was higher and HR recovery 10 min after exercise slower than in group N, which might indicate accentuated sympathetic activity after the exercise stress. The associated unpleasant feeling may be one reason why these patients feel forced to quit their previous physical activities. Increased sympathetic activation [17–21] or sympathetic excitation and parasympathetic reduction [41, 42] have been suggested to be prevalent in LC. A slow HR recovery after exercise has been reported in LC [22, 42–45], with an improvement after 5–6 months [42, 44]. An earlier study has suggested that the increased sympathetic tone might be sequelae after the viral LC infection with subintimal inflammation [46–48], leading to increased vascular stiffness, probably explaining the autonomic nervous dysfunction seen as delayed HR recovery but being reversible during follow-up [48]. Mental stress or fear may further contribute to increased sympathetic tonus [49].

Several other studies on LC have found that LC patients had reduced peak oxygen consumption (VO2) [13, 16, 23, 30, 50, 51], reduced maximal work rate [23] or ventilatory efficiency [13, 16, 34, 44, 50]. In the present study, the patients with exercise intolerance with or without fatigue had in mean a normal exercise capacity. However, we could not assess whether there was a true decrease of exercise performance because CPX results before the COVID-19 infection were not available.

In the present study, 40% of our patients had reduced exercise capacity measured as Wlast4 lower than 80% of the predicted value. Compared with Sorensen et al. [51], who found that 19% of their patients had lowered peak workload (≤ 84%), the number of patients with lowered exercise capacity is here greater. Concerning oxygen uptake % of the predicted value the results were corresponding, 50% in our study and 36% in the study by Sorensen et al. [51]. In the study by Sorensen [51], all LC patients from their clinic were tested, whereas in our study, only patients with clinical indications (e.g. cardiac symptoms, exercise intolerance, assessment of working capability etc.) were consecutively tested in our laboratory, representing a selected LC population.

It has been suggested, based on CPX results, that LC patients with reduced exercise capacity are deconditioned due to a long-term decrease in physical activity after the acute disease [52, 53]. It is obvious that also in the present patient material accentuated deconditioning explains at least some of the cases of lowered (< 80%) exercise capacity. Physical inactivity and related deconditioning are known to be associated with lower parasympathetic cardiac modulative activity and slowed HR recovery after exercise load [53–55]. Deconditioning has been shown to be resolved with exercise training [54, 55], and a recent meta-analysis found that exercise capacity measured as VO2 would improve within 3 to 6 months after the acute COVID-19 infection [24].

In the present study, several patients felt exercise intolerance despite the measured exercise capacity and oxygen uptake were within normal limits. According to our results, the explanation might be that the patients with very good physical condition would after the COVID disease be deconditioned compared to their pre-COVID felt condition. In addition, those with earlier training or other physical activities easily get worried when they feel that they are not in as good condition as before. In addition, psychological and socioeconomic factors might be a functional component in the long-lasting symptoms [56, 57].

The present study shows that CPX helps to assess possible cardiac or respiratory impairment and to identify or rule out diseases with specific treatment. Normal results are encouraging and remove obstacles to safe rehabilitation. Cardiopulmonary exercise testing is useful in assessing the patient’s exercise capacity, but not all LC patients need CPX; clinical selection of patients for referral for testing is important. The CPX results should be interpreted considering the patient’s history and physical activity in daily life. CPX results could also be used to encourage exercise if results are lowered due to inactivity and deconditioning.

The patients reported also other symptoms than fatigue and exercise intolerance. In an LC study by Contreras et al. [50], 55% of their patients reported having LC respiratory symptoms, and during exercise testing, these symptoms occurred in 44% of them. In the present study, the reported respiratory LC-symptoms occurred in 43% of the patients but restricted exercise capacity in only 9% of them. In our study, the most common causes of termination of the exercise testing included leg fatigue or discomfort (59 patients), fatigue (17 patients), and dizziness (16 patients). Breathlessness was the cause of exercise termination only in 9 patients. In comparison, in the Contreras study, 18% of patients had been acutely hospitalized compared to 10% in the present study. Additionally, 4% of their study showed slight exercise hypoxemia, whereas we did not find exercise hypoxemia. Although these are only small differences between the two studies, Contreras et al. had excluded pre-COVID respiratory diseases. However, they did not report FEV1 follow-up of their patients associated with exercise. We had 9 patients with previously diagnosed asthma in good balance, even during exercise, except one patient with increased FEV1-variation. In Contreras et al.’s study, there was a suggestion of hyperventilation during exercise testing based on an increased VE/VCO2 slope, which was not found in the present study. However, hyperventilation, according to the authors’ clinical experience and earlier literature, may start without exercise or be provoked by different methods or situations in different subjects [58]. The difference might also result from patient selection, as Contreras et al. studied dyspnea symptom, and their control subjects had some kind of dyspnea symptoms, whereas the present study focused on exercise intolerance and fatigue. Additionally, a different method of asking about the symptoms during the exercise might influence the results, but this is only speculation.

As it comes to increased sympathetic activity, there were signs of that in HR increase during exercise as well as during recovery (see Fig. 1). A POTS-type (postural orthostatic tachycardia syndrome) reaction is difficult to detect by exercise testing alone, but there was a suggestion of this, as orthostatic testing revealed an increased HR reaction in over 40% of a small cohort of the present patients.

## Strengths and limitations

The present investigation is a cohort of LC patients examined with CPX testing after being referred for testing for clinical reasons, e.g., for assessment of maximal exercise capacity or working capability or for exclusion of ischemic heart disease or respiratory causes for the symptoms. The strength of the study is that other evident diseases were already excluded before exercise testing. The reason for dealing with both exercise intolerance and fatigue followed from several patients having both symptoms. Compared to earlier studies, the present one has the benefit that it analyses and compares sympathetic overactivity, dysautonomia, and chronotropic incompetence simultaneously in one patient group and that all subjects were studied with similarly performed CPX measurements. Earlier studies have mostly analyzed them separately. In addition, in all patients included, the test was maximal according to the RER value, although in a few patients’ additional symptoms, such as chest pain and dizziness, may have contributed to the finding that the RER level > 1.1 was not reached.

In the present study, it is not possible to know if the exercise capacity of the patients had become worse after the disease because there were no earlier exercise tests available from the pre-pandemic era. Neither was a follow-up of exercise capacity measurement available.

One limitation of the study is the small size of the patient groups. In addition, the diffusing capacity measurement was conducted only in a small number of patients, as it was performed at the request of the treating physicians. The patients in the study cohort were selected by referring physicians with clinical indications to perform CPX and therefore the results cannot be generalized to represent all patients with LC.

## Conclusions

We conclude that the CPX results yield relevant information regarding LC patients suffering from subjective exercise intolerance or fatigue. In more than half of the patients, the exercise capacity was within normal limits, whereas a low HR recovery after exercise testing was found in the patients with a combination of exercise intolerance and fatigue, as suggested by somewhat increased sympathetic tonus. Those with subjective exercise intolerance tended to have the best exercise capacity. Some patients with lowered exercise capacity met the criteria for dysautonomia with increased HR at rest and during recovery phases. Although these patients had slightly lowered exercise capacity, this condition is known to be reversible. Some other subjects met the criteria of chronotropic incompetence with reduced relative HR increase, and they had the lowest exercise capacity. Although chronotropic incompetence can also be reversible, later cardiovascular diseases have been reported to be associated with this finding, and therefore, those patients should need at least a thorough follow-up of their condition. The study also indicates that subjective symptoms cannot foresee cardiopulmonary exercise capacity, and therefore CPX is needed to recognize the challenging LC patients. In addition, the results indicate that CPX is an important part of LC rehabilitation in patient selection and planning, as well as encouraging patients for self-managed rehabilitation.

### Electronic supplementary material

Below is the link to the electronic supplementary material.

Supplementary Material 1

## Data Availability

Data is provided within the manuscript or supplementary information files.

## Declarations

### Study design

This is a retrospective register study on exercise capacity and symptoms in Long Covid based on cardiopulmonary exercise testing consecutively performed between 1.6.2021–31.12.2022.

## Abbreviations

BR: Breathing reserve (measured VE related to calculated maximal ventilation)
F: Fatigue
EI: Exercise intolerance
LC: Long Covid
VO2/HR: Oxygen pulse
VO2peak: Maximal oxygen uptake
Wlast4: Maximal power during the last four minutes of exercise
Wmax/VO2peak: Work efficiency
HR: Heart rate
AT: First ventilatory threshold
VE: Minute ventilation
VE/VO2: Ventilatory equivalent for oxygen uptake
VE/VCO2: Ventilatory equivalent for CO2 production
RER: Respiratory exchange rate (VCO2/VO2)
FetCO2: Fraction of end tidal CO2
FEV1: Forced expiratory volume in one second
FVC: Forced vital capacity
