# Supplementary material for: Cardiopulmonary exercise testing in long covid shows the presence of dysautonomia or chronotropic incompetence independent of subjective exercise intolerance and fatigue
Source: BMC Cardiovasc Disord. 2024 Aug 8;24:413. doi: 10.1186/s12872-024-04081-w (PMC11308233; doi:10.1186/s12872-024-04081-w)
Supplement: Supplementary file 1 — Supplementary Material 1 [file 12872_2024_4081_MOESM1_ESM.docx]

Supplementary Table 1. The place of treatment of COVID-19 infection, LC-symptoms, and other diseases of the patients.

| **Covid-19 treatment place** |  | symptoms |
| --- | --- | --- |
| Covid-19 infection was treated at home | 87 |  |
| Covid-19 infection was treated in hospital | 10 |  |
| Covid-19 infection was treated in intensive care.  LC-symptoms in those who were treated in intensive care | 4 | 1 FA, neurological symptoms  1 palpitation, neurological symptoms  1 Combination, neurologic symptoms, and ischemia in ECG  1 with neurological symptoms, dyspnea, polyneuropathy, dysautonomia and chronotropic incompetence. |
|  |  |  |
| **Symptoms** |  |  |
| Lowered exercise capacity | 56 |  |
| Fatigue | 68 |  |
| Palpitation | 37 |  |
| Dizziness | 15 |  |
| Brain fog | 22 |  |
| Dyspnea or breathlessness | 43 |  |
| Muscle pain or discomfort | 28 |  |
| Headache | 17 |  |
| Chest pain | 33 |  |
| Neurological symptoms | 32 |  |
| Cough or fever | 6 |  |
| Gastric symptoms | 3 |  |
|  |  |  |
| **Patient groups** |  |  |
| Symptoms in group neither (group N) | 14 | chest pain and/or palpitation in 8, dyspnea or breathlessness in 4, neurological symptoms in 2 |
| Exercise intolerance (group EI) | 19 | Fatigue excluded |
| Fatigue (group F) | 31 | Exercise intolerance excluded |
| Combination (group EI+F) | 37 | both exercise intolerance and fatigue |
|  |  |  |
| **ECG-findings** |  |  |
| Group EI | 3 | p = 0.466 |
| Group F | 4 |  |
| Group EI+F | 2 |  |
| Group N | 1 |  |
|  |  |  |
| **Other diseases** |  |  |
| Depression medication | 17 |  |
| Arterial hypertension | 14 |  |
| Asthma | 9 |  |
| COPD | 1 |  |
| Migraine | 6 |  |
| Diabetes type 2 | 3 |  |
| Cardiomyopathy | 1 |  |
| Polyneuropathy | 1 |  |
| Sleep apnea | 1 |  |
| IgG deficiency | 1 |  |
| Essential thrombocytosis | 1 |  |
| Myocarditis | 1 |  |
| Spondylarthitis | 1 |  |
| Hypothyreosis | 2 |  |
|  |  |  |
| **Use of medication** |  |  |
| Astma | 13 |  |
| Beta-blockers without break | 13 |  |
| Hypertension etc., see the list above |  |  |
|  |  |  |
| **Reason for exercise termination** |  |  |
| Leg fatigue/discomfort | 59 |  |
| Fatigue | 17 |  |
| Dizziness/blurred vision | 16 |  |
| Breathlessness | 9 |  |
| Chest pain | 2 |  |
| Palpitation | 6 |  |
| ECG | 1 |  |
